# Novel Mixed Matrix Sodium Alginate–Fullerenol Membranes: Development, Characterization, and Study in Pervaporation Dehydration of Isopropanol

**DOI:** 10.3390/polym12040864

**Published:** 2020-04-09

**Authors:** Mariia Dmitrenko, Vladislav Liamin, Anna Kuzminova, Anton Mazur, Erkki Lahderanta, Sergey Ermakov, Anastasia Penkova

**Affiliations:** 1 St. Petersburg State University, 7/9 Universitetskaya nab., St. Petersburg 199034, Russia; lyamin.vlad.322@gmail.com (V.L.); ai.kuzminova@mail.ru (A.K.); a.mazur@spbu.ru (A.M.); s.ermakov@spbu.ru (S.E.); a.penkova@spbu.ru (A.P.); 2 Laboratory of Physics, Lappeenranta University of Technology, Box 20, 53851 Lappeenranta, Finland; erkki.lahderanta@lut.fi

**Keywords:** mixed matrix membrane, sodium alginate, polyhydroxylated fullerene, pervaporation, isopropanol dehydration

## Abstract

Novel mixed matrix dense and supported membranes based on biopolymer sodium alginate (SA) modified by fullerenol were developed. Two kinds of SA–fullerenol membranes were investigated: untreated and cross-linked by immersing the dry membranes in 1.25 wt % calcium chloride (CaCl_2_) in water for 10 min. The structural and physicochemical characteristics features of the SA–fullerenol composite were investigated by Fourier-transform infrared (FTIR) and nuclear magnetic resonance (NMR) spectroscopic methods, scanning electron (SEM) and atomic force (AFM) microscopies, thermogravimetric analysis (TGA), and swelling experiments. Transport properties were evaluated in pervaporation dehydration of isopropanol in a wide concentration range. It was found that the developed supported cross-linked SA-5/PAN^CaCl2^ membrane (modified by 5 wt % fullerenol) possessed the best transport properties (the highest permeation fluxes 0.64–2.9 kg/(m^2^ h) and separation factors 26–73,326) for the pervaporation separation of the water–isopropanol mixture in the wide concentration range (12–90 wt % water) at 22 °C and is suitable for the promising application in industry.

## 1. Introduction

The development of sustainable processes has drawn increasing attention worldwide. Membrane processes characterized by environmental friendliness, cost-effectiveness with low energy consumption, compact equipment, and mild operating conditions are contemporary and advanced separation technologies and can also contribute to sustainable processes. One of the most promising and actively developing membrane technologies applicable for the separation of low molecular weight substances is pervaporation. This technique is also an alternative to traditional separation methods since it allows for the separation of azeotropic and isomer mixtures, closely boiling substances, and thermally sensitive compounds by selecting a specific membrane and without using additional chemical reagents, which is complicated by conventional processes (for example, distillation or extraction) and energy-intensive. Pervaporation has received special attention for dehydration purposes (the selective removal of water from other components, namely, alcohol and solvents) [1]. The recovery of alcohols to pure form and free from traces of water is necessary due to the promising application of several alcohols as a promising alternative fuel for cars that successfully compete with gasoline in the energy market. The most common model system for membrane investigation in pervaporation dehydration is the isopropanol–water mixture that contains an azeotrope (12 wt % water–88 wt % isopropanol) [2]. The separation by conventional methods of this system requires the additional separating agent of benzene or cyclohexane (to break the azeotropic mixture) with the subsequent distillation. With such a strategy, it is almost impossible to separate cyclohexane from alcohol. However, it is necessary to use membranes based on biopolymers with tailored transport characteristics to further develop pervaporation as a sustainable process. This work is devoted to the development of pervaporation membranes based on sodium alginate.

Sodium alginate (SA) is an important biopolymer derived from vegetable sources (from algae by treatment with NaOH). This polymer is actively used in the biomedical field (for the production of drug delivery systems, in wound dressings and tissue regeneration) [3,4]; food industry (thickeners and gelling agents in foodstuffs); textiles and paper industries [5]; cosmetics as alginate masks, etc. due to its high biocompatibility, solubility in water, low toxicity, ability to form stable aqueous hydrogels, and film-forming properties. Sodium alginate is also actively applied in membrane technology as a polyelectrolyte [6,7,8] and for the preparation of membranes for fuel cell applications [9], vapor permeation [10,11,12], nanofiltration [13,14], ultrafiltration [15], and pervaporation [16,17,18,19,20].

One of the main disadvantages of pristine SA membranes for pervaporation dehydration is the relatively low stability of the membranes, especially in feed solutions with a high water content. To improve physicochemical and transport properties of the SA membranes, various efforts are applied by cross-linking of the membranes [19,21,22], chemical modifications [5], blending with other polymers [14,23,24,25], and the development of mixed matrix membranes (MMMs) (the formation of organic–inorganic composites). Modifiers such as metal oxides [21,26,27,28], attapulgite nanorods [19], covalent organic frameworks [29,30], glycogen [22], zeolites [18,31], metal–organic frameworks [20,32,33,34,35], etc. have been used to develop hybrid SA membranes. In particular, one of the most promising directions of polymer modification (in the development of MMMs) is the introduction of carbon nanoparticles into the polymer matrix. In earlier studies, their relevance and improvement of the pervaporation characteristics were demonstrated for the polymer membranes based on polysulfone [36], polyphenylene oxide [37,38], polyphenylene isophthalamide [38,39,40], and polyvinyl alcohol [41,42,43]. Despite the prospects of this direction, the literature review has demonstrated that there are a limited number of works on the modification of SA membranes by carbon nanoparticles, namely, SA modification has been carried out only by graphene, graphene oxide, and functionalized multiwalled carbon nanotubes. The authors of [44,45] used modified (decorated by magnetite (Fe_3_O_4_) nanoparticles and chitosan-wrapped, respectively) multiwalled carbon nanotubes (MWCNT) as fillers for SA membranes for pervaporation dehydration. The supported (on the polyacrylonitrile porous substrate) SA/MWCNT-Fe_3_O_4_ (2 wt %) membrane exhibited excellent separation performance for ethanol dehydration (10 wt % water) at 76 °C: 2.2 kg/(m^2^ h) and 1870 separation factor [44], while dense SA membrane containing 2 wt % of CS-wrapped MWCNT demonstrated 6419 separation factor and 0.23 kg/(m^2^ h) permeation flux for pervaporation separation of water–isopropanol (10/90 wt %) at 30 °C [45]. Suhas et al. developed graphene-loaded sodium alginate nanocomposite membranes for pervaporation isopropanol dehydration [46]. At the lowest concentration of graphene (2 wt %) the hybrid membrane separation performance was optimum (permeance value of 3122 GPU and separation factor of 4623) for separation of water–isopropanol (10/90 wt %) at 30 °C. In [47], pristine graphene oxide (pGO) and reduced graphene oxide (rGO) nanosheets were incorporated into the sodium alginate matrix. It was shown that rGO-filled (1.6 wt %) membranes exhibited improved separation factor (1566) and an unusual change in permeation flux (1.7 kg/(m^2^ h)) in pervaporation dehydration of 90 wt % ethanol aqueous solution at 76 °C. In [48], graphene oxide quantum dots (GOQDs) were used as a modifier to develop a novel nanocomposite SA membrane for ethanol dehydration. The obtained nanocomposite membrane possessed improved permeation flux (2.4 kg/(m^2^ h)), which was increased by 60% compared with the pristine SA membrane and 1152 separation factor in pervaporation dehydration of 90 wt % ethanol aqueous solution at 77 °C. The authors in [49] developed a SA membrane for efficient water permeation and water/alcohol separation by the incorporation of zwitterionic graphene oxides (PSBMA@GO) nanosheets into the polymer matrix. The MMMs with PSBMA@GO (2.5 wt %) exhibited much higher separation performance (2.1 kg/(m^2^ h) permeation flux and 1370 separation factor in the pervaporation of 90 wt % ethanol aqueous solution at 77 °C) compared with membranes based on pure SA and on a composite with unmodified GO. Thus, the previous studies demonstrated the prospects of carbon nanoparticle application as modifiers for the development of hybrid SA membranes with improved characteristics. However, it should be noted that there is no information on SA modification by fullerene and its water-soluble derivatives, although this modification can significantly improve the transport properties of polymer membranes [42,43,50,51,52].

The aim of the present work was to study the effect of the introduction of polyhydroxylated fullerene (fullerenol) to SA on the transport and physicochemical characteristics of membranes. This carbon particle was chosen due to the presence of polar groups (hydroxyl groups –OH). These groups are expected to contribute to better dispersion of fullerenol in the hydrophilic polymer matrix and to lead to surface membrane functionalization and cross-linking of polymer chains during the modification process. Two types of SA-based membrane containing fullerenol were developed: dense and supported (composite) membranes. Supported membranes were developed to increase the permeation flux due to the formation of a thin dense selective layer on a porous commercial substrate from polyacrylonitrile, which provided mechanical strength. The developed membranes were used without additional treatment and by applying cross-linking by immersing the dry membranes in 1.25 wt % calcium chloride (CaCl_2_) in water for 10 min. The structural and physicochemical characteristics of the SA–fullerenol mixed matrix membranes were investigated by Fourier-transform infrared (FTIR) and nuclear magnetic resonance (NMR) spectroscopic methods, scanning electron (SEM) and atomic force (AFM) microscopies, thermogravimetric analysis (TGA), and swelling experiments. Transport properties were evaluated in the pervaporation dehydration of isopropanol in a wide concentration range. However, it should be noted that the developed membranes based on SA may be applied in any pervaporation dehydration process. 

## 2. Materials and Methods 

### 2.1. Materials

Sodium alginate (SA) with the viscosity of 0.09 Pa∙s in the form of powder (manufactured by Jiangsu Benefit Ocean Technology Co. Ltd., Lianyungang, China) obtained from OOO “BIOPROD” (St. Petersburg, Russia) was used as the membrane material. Polyhydroxylated fullerene (fullerenol) C_60_(OH)_22–24_ (Fullerene Technologies, St. Petersburg, Russia) was used for SA modification. Isopropanol (i-PrOH) (Vekton, St. Petersburg, Russia) and the chemical for the cross-linking of membranes, calcium chloride (CaCl_2_), were used without additional treatment.

A hydrophilic commercial porous membrane based on polyacrylonitrile (PAN, Institute “Leibniz-Institut für Polymerforschung Dresden”, Dresden, Germany) was used as the membrane substrate for the preparation of supported membranes with a thin selective top layer.

### 2.2. Membrane Preparation

#### 2.2.1. Dense Membranes

The membranes were prepared according to the following procedure: 1 wt % SA solution was prepared by dissolving a determined amount of the polymer in water and stirring for 4 h at 45 °C. Then, fullerenol dispersion (0–10 wt % of C_60_(OH)_22–24_ with respect to the polymer weight) was added to the SA solution, followed by ultrasonic treatment at ambient temperature. Dense membranes were prepared by pouring a polymer solution or polymer/fullerenol composite into a Petri dish followed by solvent evaporation in an oven at 40 °C for 24 h. The maximal concentration of fullerenol was 10 wt % in the polymer matrix, as above this concentration, it resulted in poor dispersion of fullerenol into the polymer matrix and membrane defectiveness. The dense membrane thickness was measured by a micrometer and was found to be 25 ± 3 μm.

#### 2.2.2. Supported Membranes

To increase the permeability of dense membranes, supported membranes with a thin dense selective layer deposited on the porous substrate were prepared by casting SA solution or its SA/fullerenol (5%) composite onto the surface of the porous substrate based on polyacrylonitrile (PAN) and drying at room temperature for 24 h to evaporate the solvent. The application of the PAN substrate decreased the thickness (to 600 ± 100 nm) of the top selective polymer layer, increasing membrane permeability and providing the mechanical strength of this thin dense layer, which was prepared according to the procedure described for dense membranes.

**Figure 1:**
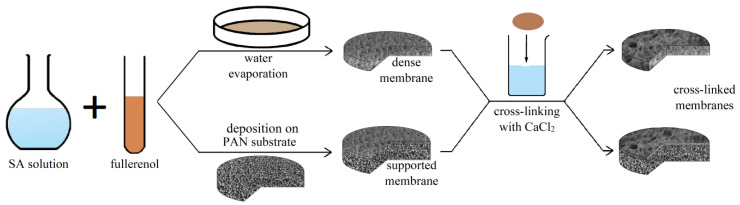
The scheme of preparation and cross-linking of dense and supported membranes based on sodium alginate (SA) and its composite SA–fullerenol.

The developed dense and supported membranes based on SA and the SA/fullerenol composite were used without additional treatment (untreated) and were subjected to chemical cross-linkage for the application of membranes for the pervaporation separation of more diluted solutions. The most commonly used cross-linking method [26,53] was applied: immersing the dry membranes in 1.25 wt % calcium chloride (CaCl_2_) in water for 10 min. All cross-linking treatments were carried out at ambient temperature with the subsequent washing of cross-linked membranes with deionized water. Figure 1 demonstrates schematically the methods for preparation and cross-linking of dense and supported membranes based on the polymer SA and its composites with fullerenol. 

The denotations of developed SA membranes and the preparation conditions are given in Table 1. To simplify the designation of membranes, the fullerenol concentrations (3, 5, 7, and 10 wt %) are indicated as a number, the applied commercial PAN substrate for the preparation of supported membranes is written through a slash and an abbreviation of an additional cross-linking agent is indicated in the form of a superscript.

### 2.3. Fourier Transforms Infrared Spectroscopy (FTIR)

To determine the structural changes during the modification of SA by fullerenol, FTIR spectroscopy was performed on a spectrometer—IRAffinity-1S (Shimadzu, St. Petersburg, Russia) using an attenuated total reflectance (ATR) accessory (PIKE Technologies, Moscow, Russia) in the range of 649–4000 cm^−1^ at 25 °C with 40 scans and a resolution of 2 cm^−1^.

### 2.4. Nuclear Magnetic Resonance (NMR)

NMR study of the membranes was conducted with a Bruker Avance III 400 WB NMR spectrometer (Bruker, Ettlingen, Germany) (magnetic field of 9.4 T) using a 4-mm CP/MAS probe. Larmor frequency for investigated nuclei ^13^C was 100.64 MHz. Samples were loaded into a zirconium oxide 4 mm rotor spun at 12.5 kHz. Liquid tetramethylsilane (TMS) was used as an external reference for ^13^C nuclei. {^1^H}^13^C CP/MAS NMR spectra were acquired with 8192 scans, 2 ms contact time, and 5 s relaxation.

### 2.5. Scanning Electron Microscopy (SEM)

The inner structure of the prepared SA membranes was studied by scanning electron microscopy (SEM) with Zeiss Merlin SEM (Carl Zeiss SMT, Oberhochen, Germany). The membranes were not sputtered to keep the sample’s surface morphology unchanged and were observed using low accelerating voltage of 1 kV and a low electron beam current of 100 pA to prevent surface charging and modification during SEM imaging. The membrane was submerged in liquid nitrogen for about one minute and was fractured perpendicular to the surface in the liquid nitrogen after reaching the Leidenfrost point [54]. After crushing, the sample was removed from liquid nitrogen and dried in air for 5 min.

### 2.6. Atomic Force Microscopy (AFM)

NT-MDT NTegra Maximus atomic force microscope (NT-MDT Spectrum Instruments, Moscow, Russia) with standard silicon cantilevers with a rigidity of 15 N·m^−1^ in tapping mode was used to study the surface topography of the SA membranes. 

### 2.7. Thermogravimetric Analysis (TGA)

To evaluate the thermochemical properties of the membranes, the thermogravimetric analysis (TGA) was carried out using a Thermobalance TG 209 F1 Libra (Netzsch, Leuna, Germany) (resolution of 0.1 μg over the entire weighing range, balance drift—less than 5 μg/hour). Samples (~2 mg) were placed in the pans of the TGA instrument and analyzed over the temperature range 37–720 °C with the heating speed of 10 °C/min under an inert argon atmosphere.

### 2.8. Swelling Measurement

The equilibrium swelling degree (sorption) was studied in water and the azeotropic water–isopropanol mixture by a gravimetric method at 20 °C for untreated and cross-linked membranes based on SA and SA–fullerenol composite. Membranes of known weight were immersed in weighing bottles containing water or the azeotropic water–isopropanol mixture and were weighted from time to time until the weight of the swelling films remained constant.

The swelling degree (sorption) (S) was calculated by the following Equation (1):

(1) $$S=\frac{m_{s}-m_{o}}{m_{o}}$$

where *m*_s_ (g) is the weight of a swollen membrane and *m*_o_ (g) is the weight of a dry membrane.

### 2.9. Pervaporation Experiment

**Figure 2:**
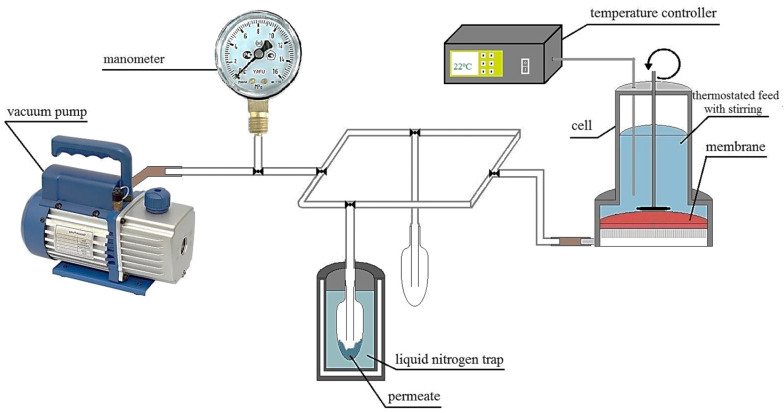
The scheme of the pervaporation setup.

The transport properties of the developed membranes were studied using a laboratory cell in the steady-state regime at 22 °C in the stirring regime (Figure 2). The compositions of permeate and feed were analyzed by gas chromatography using Chromatek Crystal 5000.2 (Chromatec, Nizhny Novgorod, Russia) gas chromatograph equipped with the “Hayesep R” column and a thermal conductivity detector.

The permeation flux J (kg/(m^2^ h)) of membranes was determined to be the amount of liquid transported through a unit of the membrane area per hour and was calculated as [55]:

(2) $$J=\frac{W}{A\cdot t}$$

where *W* (kg) is the component weight that permeated the membrane; *A* (m^2^) is the effective membrane area; and *t* (h) is the measurement time.

The separation factor (*β*), the pervaporation separation index (PSI), and component permeances (P/l) were calculated to evaluate the effectiveness of pervaporation separation of the isopropanol–water mixture using developed SA membranes. 

The separation factor (*β*) was calculated as [56]:

(3) β=yiyjxixj

where *y_i_* and *y_j_* are the weight fractions of components *i* and *j* in the permeate and *x_i_* and *x_j_* are the weight fractions of components *i* and *j* in the feed.

The pervaporation separation index (PSI) was calculated as:

(4) PSI=J·(β−1)

The permeance P/l is a component flux normalized for the driving force calculated according to the work of R.W. Baker et al. [56] as:

(5) $$\frac{P}{l}=\frac{j_{i}}{p_{i_{f}}-p_{i_{p}}}$$

where *j_i_* is the partial flux of the *i* component; *l* is the membrane thickness; and $p_{i_{f}}$ and $p_{i_{p}}$ are the *i* component vapor pressures of the feed and the permeate, respectively. The component permeances were expressed in common gas permeation units (GPU) (1 GPU = 1 × 10^−6^ cm^3^ (STP)/cm^2^ s cm Hg; 1 m^3^ m/m^2^ s kPa = 1.33 × 10^8^ GPU).

Each measurement was performed at least three times to ensure good accuracy of the transport parameters, and the average value was recorded for later analysis. The mean accuracy for the transport parameters was as follows: ±0.4% for water content in the permeate and ±8% for permeation flux for the dense membranes; ±0.3% for water content in the permeate and ±5% for permeation flux for the supported membranes.

## 3. Results

The effect of fullerenol introduction into the SA matrix on the membrane transport properties was studied in the pervaporation dehydration of isopropanol. Two types of membranes were developed: dense and supported. These mixed matrix membranes were characterized by different methods of analysis. The obtained data are presented in the current section.

### 3.1. The Development and Investigation of Dense Membranes

#### 3.1.1. Pervaporation Performance of Dense SA Membranes

Pervaporation experiments for SA membranes were carried out for the separation of the water–isopropanol mixture. As pervaporation is described by a solution–diffusion mechanism (three stages: sorption, diffusion, and desorption), it is necessary to evaluate the interaction and affinity between SA and feed components of various polarity (water and isopropanol). One of the most widely-used and fast approaches is the use of Hansen’s Solubility Parameters including the determination of three major types of the intermolecular interactions: dispersion (δ_d_), polar (δ_p_), and hydrogen bonding (δ_h_) [57], which affects membrane diffusion and permeability. The total solubility parameter (δ_t_) can be calculated from these parameters [58]. Literature data on the Hansen’s solubility parameters for SA, water, and isopropanol were used to evaluate the compatibility of SA to the feed components (Table 2).

The value of distance parameter (∆) is required to evaluate the affinity between two components (polymer and solvent) using the partial solubility parameters of pure solvents and polymer [60]. The δ_d_ values for water and isopropanol are relatively the same, while δ_p_ and δ_h_ differ considerably (Table 2). This indicates that the interactions between SA and the solvent mainly depend on the parameters of the polar and hydrogen bonds, while the dispersion parameter almost does not affect these interactions [60]. In the literature, we could locate the data only for the total solubility parameter for SA [58], and there were no calculations and estimations for individual parameters δ_d_, δ_p_, and δ_h_. Nevertheless, the total parameters of SA and solvents could be evaluated. The relative similarity of the total parameters of solvent and SA determines the potential polymer dissolution in the solvent and, at the same time, a higher degree of the solvent’s sorption in the polymer [57,61]. ∆δ_t_ for SA and water (10.8) is lower compared with isopropanol (∆δ_t_ = 13.4), which suggests that the SA membrane has a high affinity to water and would exhibit the highest selectivity properties (confirmed by the pervaporation data). It is also worth noting that water’s larger δ_h_ parameter compared to isopropanol is the most important factor affecting the solubility of SA with ionized COOH groups [58] (confirmed by swelling data, Table 5). 

Pervaporation experiments for untreated SA membranes were carried out for the separation of azeotropic water (12 wt %)—isopropanol (88 wt %) mixture because of the low stability of non-cross-linked membranes in aqueous solutions. The application of the cross-linking method for SA and SA–fullerenol membranes allows using them for pervaporation separation of the water-isopropanol mixture in a wide concentration range (12–100 wt % water in the feed). This was performed to test the performance and stability of the developed membranes in the presence of water excess, which could easily occur in industrial processes.

**Figure 3:**
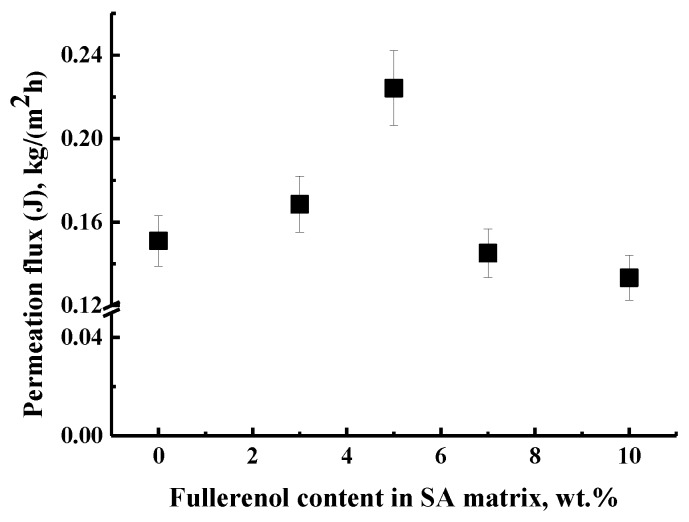
The dependence of permeation flux on fullerenol content in the SA membrane in pervaporation of azeotropic water (12 wt %)—isopropanol (88 wt %) mixture at 22 °C. Water content in the permeate for all membranes was 99.99 wt %.

The transport properties of untreated dense SA and SA–fullerenol (3, 5, 7, 10 wt %) membranes in the pervaporation separation of azeotropic water–isopropanol (12/88 wt %) mixture at 22 °C are presented in Figure 3.

It was demonstrated that all SA membranes were highly selective with respect to water (99.99 wt % water content in the permeate), while permeation flux significantly increased up to 5 wt % fullerenol in the SA matrix (SA-5 membrane) and decreased for the SA-7 and SA-10 membranes (Figure 3). This effect was also confirmed by the NMR data for SA-based membranes (NMR crystallinity data, Table 3), where it was shown that the introduction of 5 wt % fullerenol into the SA matrix led to structuring of the SA membrane, which contributed to an increase in the permeability of the SA-5 membrane. The decrease in the permeation flux for SA-7 and SA-10 membranes to a greater extent can be associated with the existence of an impenetrable volume due to the presence of agglomerates of carbon modifiers that created an impenetrable space for penetrants through the SA-7 and SA-10 modified membranes (shown by SEM data, Figure 7d,e). 

Based on the obtained pervaporation data for the separation of the azeotropic water–isopropanol mixture, it was demonstrated that the SA membrane modified by 5 wt % fullerenol (SA-5) possessed the best transport characteristics (the highest permeation flux). Thus, to apply this membrane in the pervaporation separation of water–isopropanol mixture in a wide concentration range, SA-5 and SA-0 membranes were cross-linked by immersing in 1.25 wt % calcium chloride (CaCl_2_) aqueous solution for 10 min. The membrane based on pristine polymer was also cross-linked for a comparison with the properties of the modified SA-5^CaCl2^ membrane.

**Figure 4:**
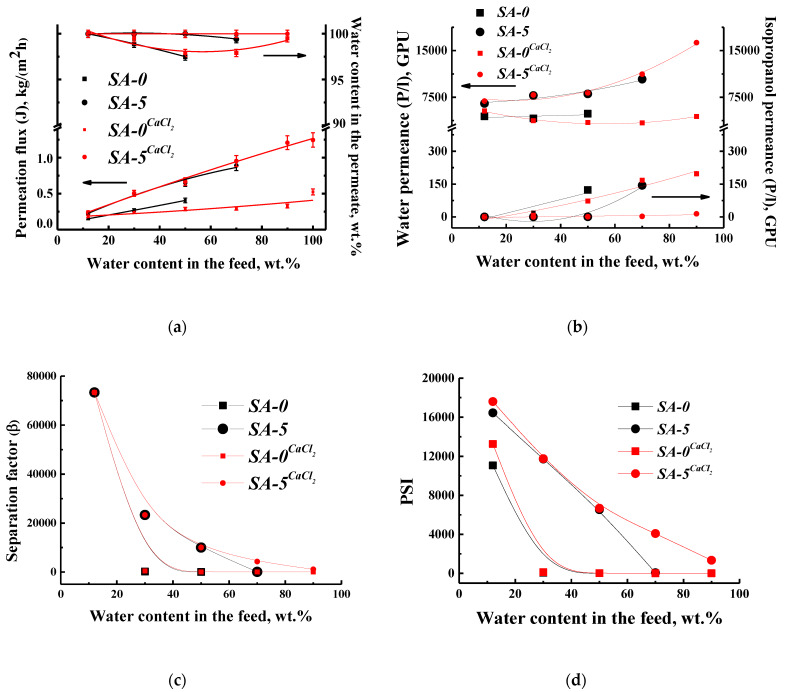
The dependence of (**a**) permeation flux and water content in the permeate, (**b**) water and isopropanol permeances, (**c**) separation factor, and (**d**) pervaporation separation index (PSI) on water content in the feed during pervaporation of the water–isopropanol mixture at 22 °C for untreated and cross-linked SA and SA–fullerenol (5%) membranes.

The transport properties for untreated and cross-linked by calcium chloride SA and SA–fullerenol (5%) membranes (SA-0^CaCl2^ and SA-5^CaCl2^) (i.e., permeation flux, water content in the permeate, component permeances, separation factor, and pervaporation separation index (PSI)) in the pervaporation of a water–isopropanol mixture (12–100 wt % water) are presented in Figure 4.

It was demonstrated that the untreated SA-0 membrane was applicable for the pervaporation separation to 50 wt % water in the feed, while the introduction of 5 wt % fullerenol into the SA matrix (SA-5) allowed for this membrane to be applied to up to 70 wt % water in the feed, which also confirmed the cross-linking effect of SA by fullerenol (in accordance with the swelling degree data, Table 5). For higher water concentrations, the SA-0 and SA-5 membranes lost integrity (Figure 4a). The cross-linking by CaCl_2_ of the membranes made it possible to use membranes over the entire concentration range of the water–isopropanol mixture (12–90 wt % water) as well as to test these membranes for pure water penetration (Figure 4a). Additionally, the cross-linking of the SA membrane (SA-0^CaCl2^) led to an insignificant decrease in the permeation flux and an increase in selectivity (higher water content in the permeate) compared to the untreated SA-0 membrane. The cross-linked membrane modified with fullerenol (SA-5^CaCl2^) had an increased permeation flux and water content in the permeate compared to the cross-linked SA-0^CaCl2^ and the untreated SA-5 membranes (Figure 4a) due to structural and morphology changes (SEM, AFM data, Figure 8). This effect is related to the fact that fullerenol also acts as a modifier and a cross-linker of the SA membrane and eliminates the cross-linking effect of calcium chloride, which was also confirmed by NMR data.

The pervaporation performances of the developed membranes were also represented in terms of component permeances, separation factor, and PSI in Figure 4b,c,d, respectively. It was shown that water permeance followed the same trend, demonstrating the highest values for the SA-5^CaCl2^ membrane, while the isopropanol permeance was minimal for this membrane (Figure 4b). The values of the separation factor and PSI for the SA-5^CaCl2^ membrane were close to the SA-5 membrane up to 50 wt % water in the feed, and after increased, demonstrating the better transport properties of the cross-linked modified membrane (Figure 4c,d). Thus, the developed cross-linked SA membrane modified by 5 wt % fullerenol (SA-5^CaCl2^) had the optimal transport properties (the highest permeation flux and selectivity) for the pervaporation separation of the water–isopropanol mixture in a wide concentration range. However, a further increase in performance is required for application in industry, which can be achieved by the development of the supported membranes presented in Section 3.2. The change of transport characteristics of SA-based membranes after modification and cross-linking can be explained by changes in structure, morphology, and surface topography, which were investigated by a different method of analysis and presented in Section 3.1.2.

#### 3.1.2. Structure and Physicochemical Properties Investigation

The structural characteristics of untreated and cross-linked SA membranes modified by fullerenol were studied by FTIR and NMR spectroscopies as well as scanning electron (SEM) and atomic force (AFM) microscopies.

**Figure 5:**
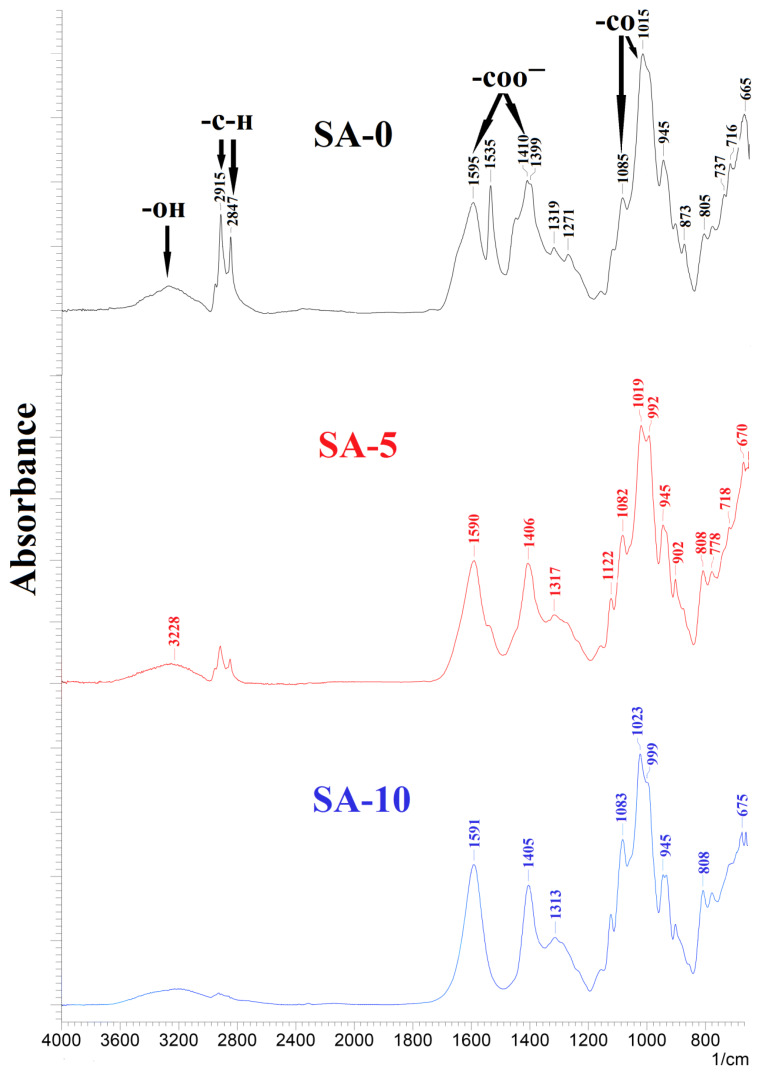
The Fourier transform infrared (FTIR) spectra for the SA-0, SA-5, and SA-10 membranes.

FTIR spectra for untreated membranes based on sodium alginate (SA-0) and sodium alginate–fullerenol (5 or 10 wt %) (SA-5 and SA-10) are presented in Figure 5.

The FTIR spectrum of the unmodified SA-0 membrane demonstrated primary absorption bands at 3228, 1595, 1410 cm^−1^, 2914–2845, and 1020–1095 cm^−1^, which corresponded to the stretching of the hydroxyl group, symmetric and asymmetric stretching of the carboxylate group, and the stretching of aliphatic C–H and C–O bonds, respectively [26,34,62,63]. After the introduction of 5 wt % fullerenol into the SA matrix, some characteristic band changes were observed: (1) the decrease in peak intensity at 3228 cm^−1^ was assigned to the reduction of –OH groups, (2) the significant decrease of peak intensity at 2915, 2847 (corresponding to the stretching of aliphatic C–H and C–O bonds), and 1535 cm^−1^, and (3) the shift peaks at 1595 and 1085 cm^−1^ to the to lower wavenumbers (1590 and 1082 cm^−1^) compared with the spectrum of the pure SA membrane may be related to the overlapping peaks of sodium alginate and fullerenol characteristic peaks (corresponding to stretching of C=C and C–O bonds [64]). These changes in the peak positions and relative intensities increased with increasing fullerenol content in the SA membrane from 5 to 10 wt %. This may indicate that the formation of the hydrogen bonds between the fullerenol and SA caused strong interfacial interactions [65].

**Figure 6:**
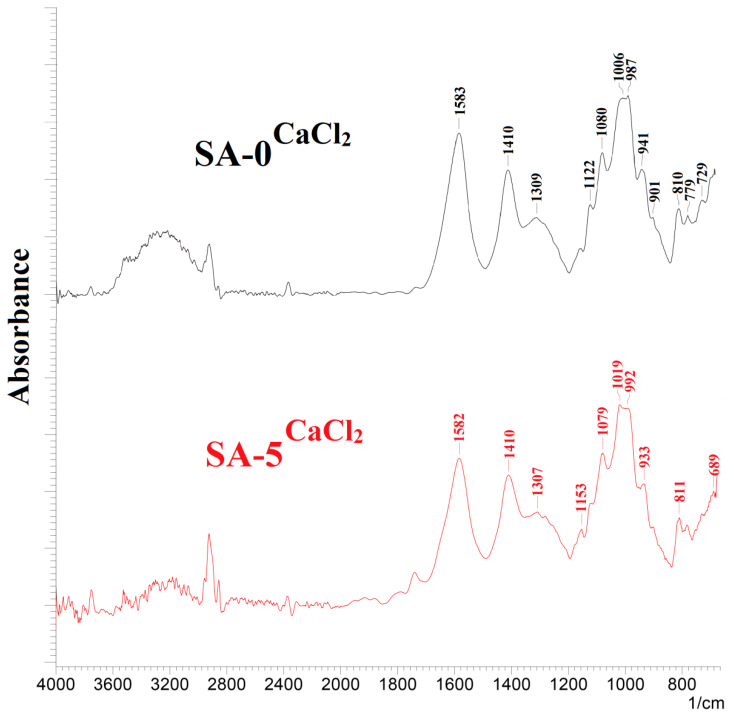
The FTIR spectra for the SA-0^CaCl2^ and SA-5^CaCl2^ membranes.

The spectra of cross-linked membranes based on SA and composite SA–fullerenol (5%) (SA-0^CaCl2^ and SA-5^CaCl2^) are presented in Figure 6. Through FTIR spectroscopy, it was shown that the cross-linking by CaCl_2_ did not significantly influence the structural properties of SA membranes. The cross-linking of SA-0 with calcium cations led to the increased hydrophilicity of the membranes (which is expressed by an increase in the extension band assigned to –OH groups) (Figure 6) and to the ionic interaction of polymer chains with Ca^2+^ with the formation of electronegative cavities [26], resulting in the cross-linking of SA chains in the structure of an “egg box” [53]. This was also confirmed by the differences in spectrum bands: the peak shift to 1583 cm^−1^ (corresponding to the asymmetric and symmetric stretching of –COO^−^ associated with carboxylic acid salts) and the significant increase in the peak intensity at 1085 cm^-1^ (related to the C–C and C–O stretching) for the SA-0^CaCl2^ membrane, which could also be attributed to the presence of the cross-linking of polymer chains [66].

The FTIR spectra for the SA-0^CaCl2^ and SA-5^CaCl2^ membranes showed no significant changes in the structure for the SA-5^CaCl2^ membrane (modified by 5 wt % fullerenol and cross-linked with calcium chloride) compared to the SA-0^CaCl2^ membrane, in addition to a significant reduction in the band assigned to the –OH groups. 

Through the NMR method, it was shown that the spectra of the developed membranes corresponded to previously published spectra of sodium alginate [67], which was a linear copolymer consisting of homopolymer blocks of munnuronate (M) and guluronate (G) (Figure S1) [68]. The ^13^C CP/MAS NMR spectra of the untreated and cross-linked SA and SA–fullerenol membranes are presented in Figure S2. The peak in the range of 170–185 ppm (corresponding to carbon atoms under number 6 in the structure of SA (Figure S1)) was characterized by inhomogeneous broadening in the spectra for those cross-linked by CaCl_2_ membranes when compared to the spectra of untreated membranes. These changes in spectra related to the cross-linked polymer blocks around calcium ions. However, the introduction of fullerenol into the SA matrix led to a decrease in the cross-linking effect of calcium chloride and reduced the linked number of polymer blocks. In the spectral range of 91–108 ppm (corresponding to carbon atoms under number 1 in the structure of polymer (Figure S1)), an inhomogeneously broadened spectral line was observed for the developed membranes. Through analogy with the results of the NMR studies of cellulose samples, the presence of amorphous and crystalline phases in the membranes in this range can be assumed [69]. It should be emphasized that crystallinity calculated from the NMR data determines the contributions from chain segments, which are included in small and defective crystals and is based on segment conformations or packing regularity without the long-range order required for wide angle X-ray diffraction (WAXD) analysis [70]. Moreover, the values of crystalline phase content from the NMR data are often much higher than for WAXD data because of the additional inclusion of crystallites that are too small and disordered to contribute to crystalline diffraction [70]. The lines in the range of 91–108 ppm were decomposed into two components to analyze the quantitative change of these phases and was estimated according to the area ratio of the received lines components percentage ratio (crystalline phase percentage). The crystalline phase content for untreated and cross-linked SA and SA–fullerenol (5%) membranes is presented in Table 3.

The untreated and cross-linked membranes should be considered separately in terms of different mechanisms of formation and, as a consequence, of the explanation of the obtained data.

For the untreated membranes, the data in Table 3 demonstrate that the fraction of the crystalline phase sharply increases with the introduction of 5 wt % fullerenol into the SA matrix (for SA-5 membrane), and again decreases for the SA-10 membrane. An increase in crystallinity for the untreated SA-5 membrane means structuring of the SA matrix by fullerenol, creating crystalline phases near the modifier molecule, which contributes to the more amorphous state of the membrane matrix. This effect caused increased permeability for untreated SA-5 membrane compared to the pristine SA-0 membrane. 

In the case of the cross-linking of SA membranes by calcium chloride, the polymer chains were formed in the structure of an “egg box” [53,66] (confirmed by FTIR data, Figure 5 and Figure 6). Thus, cross-linked SA-0^CaCl2^ membrane becomes stable in water (83% swelling degree in water, Table 5) and possesses lower permeation flux compared to the untreated SA-0 membrane (Figure 4) due to the strong cross-linking effect and rigid structure (TGA data, Figure 9). The introduction of fullerenol eliminates the effect of cross-linking and structuring of the SA matrix by calcium chloride for the SA-5^CaCl2^ membrane due to the fact that fullerenol itself acts as a cross-linker. This is reflected in the decrease of the crystalline phase content for this membrane when compared to the SA-0^CaCl2^ membrane (Table 3), resulting in the significant increase in permeability for the modified membrane. At the same time, it retains the cross-linking structure of the membrane. The confirmation of this effect has also been noted by the wide unresolved spectral lines in the range of 58–90 ppm, which corresponds to carbon atoms under numbers 2–5 in the structure of SA, where the position of the peaks for the blocks of munnuronate (M) and guluronate (G) can be significantly different [67]. By changing the peak positions that correspond to carbon atoms in the same SA structural positions (Table S1), it can be concluded that the largest changes are observed for peaks G4 and G5 of the cross-linked membranes, which may correspond to the participation of these blocks in the cross-linking mechanism. At the same time, for the cross-linked SA-0^CaCl2^ membrane, the peak corresponding to carbon atoms in the M5 position shifts strongly, which indicates that the units of munnuronate (M) can also participate in the cross-linking mechanism. However, for the SA-5^CaCl2^ membrane (the introduction of 5 wt % fullerenol into the SA matrix and cross-linking by CaCl_2_), the blocks of munnuronate (M) are practically excluded from this mechanism, which indicates a decrease in the effect of cross-linking with calcium chloride due to the presence of fullerenol.

**Figure 7:**
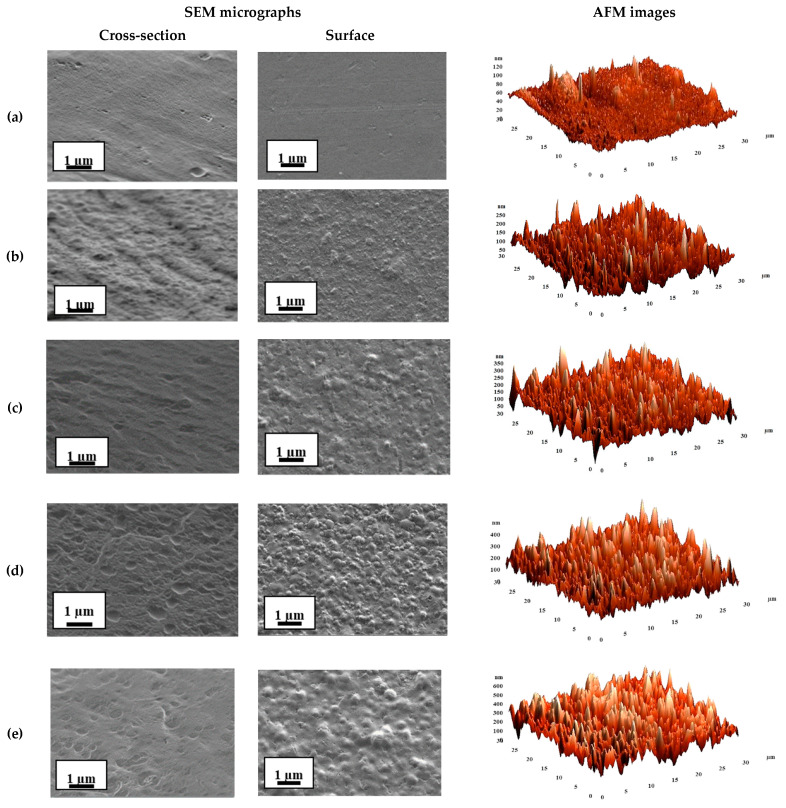
The cross-sectional and surface SEM micrographs and AFM images of the SA-based membranes: (**a**) SA, (**b**) SA-3, (**c**) SA-5, (**d**) SA-7, (**e**) SA-10.

**Figure 8:**
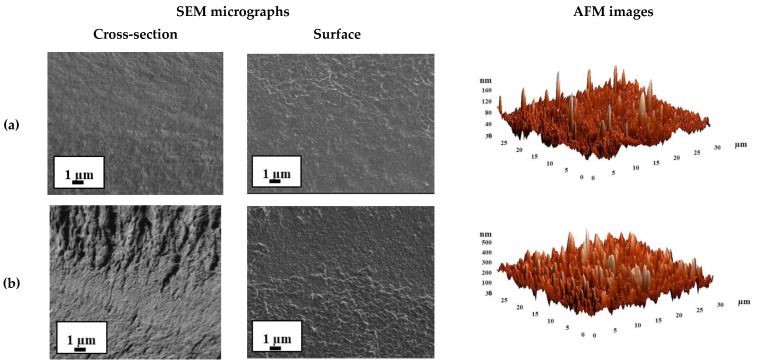
The cross-sectional and surface SEM micrographs and AFM images of cross-linked (**a**) SA-0^CaCl2^ and (**b**) SA-5^CaCl2^ membranes.

The inner morphology and the surface topography of the untreated and cross-linked membranes based on SA and its composite with fullerenol were studied by SEM and AFM. The results are presented in Figure 7 and Figure 8, respectively.

The cross-sectional and surface SEM micrographs and AFM images with a scan size of 30 × 30 μm for the untreated SA and modified membranes are presented in Figure 7.

The SEM micrographs (Figure 7) demonstrated that the cross-sectional and surface structure of dense SA membrane significantly changed with the fullerenol introduction into the SA matrix. The membrane based on pristine SA possessed a smooth and plain structure in the cross-section and surface, while the modification of the SA membrane by fullerenol led to the appearance of the plastic deformations in the form of “knolls” on surfaces and “grooves” on the cross-sections, which significantly increased with the rise of fullerenol content in the SA matrix. All these modifications can provide significant changes in transport properties when compared to a pristine SA membrane.

For the comparison with the untreated membranes, the inner morphology and the surface topography of cross-linked SA-0^CaCl2^ and SA-5^CaCl2^ membranes were also studied by SEM and AFM. The cross-sectional and surface SEM micrographs and AFM images with a scan size of 30 × 30 μm for SA-0^CaCl2^ and SA-5^CaCl2^ membranes are presented in Figure 8.

It was demonstrated that the cross-linking of SA-0 and SA-5 membranes by calcium chloride (CaCl_2_) led to the significant changes in the inner and surface morphology of the membranes. The SA-0^CaCl2^ membrane had a pronounced roughness structure of the cross-section and surface (Figure 8a) when compared with the untreated SA-0 membrane (Figure 7a). The modification of SA by 5 wt % fullerenol and cross-linking by CaCl_2_ greatly enhanced the asymmetric roughness and heterogeneity of the SA-5^CaCl2^ membrane morphology. 

The roughness characteristics of the SA and SA–fullerenol membrane surfaces were calculated based on AFM images (Figure 7 and Figure 8) in terms of the root-mean-squared surface roughness (R_q_) and average roughness (R_a_) (Table 4), these characteristics may strongly affect the sorption of the separated feed components on the membrane surface during pervaporation separation and the membrane permeability. 

It was demonstrated that the introduction of fullerenol into the SA matrix led to the significant increase of the surface roughness of membranes. The values of R_a_ (average surface roughness) for the untreated hybrid membranes were 3.5–9.4 times higher when compared to the average surface roughness of the pristine SA membrane (7.2 nm) (Table 4). The application of cross-linking by CaCl_2_ for SA and SA–fullerenol (5%) membranes (SA-0^CaCl2^ and SA-5^CaCl2^) also led to an increase in the surface roughness R_a_ of 1.6 and 1.4 times when compared to the SA-0 and SA-5 membranes, respectively. It provided a larger surface membrane area for contact with the separated mixture, leading to facilitated sorption of feed components and faster penetration of the components through the membrane, resulting in a significant improvement in membrane permeability. Additionally, the increase in the surface roughness of membranes was in accordance with the SEM data.

According to SEM, AFM, and the pervaporation data, during the modification of SA membranes, fullerenol was evenly distributed in bulk and on the surface of the SA membranes. 

**Figure 9:**
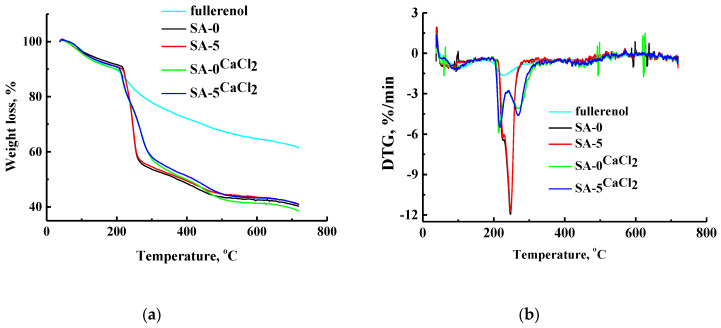
(**a**) TG and (**b**) DTG curves for fullerenol, SA-0, SA-5 and cross-linked SA-0^CaCl2^ and SA-5^CaCl2^ membranes.

Thermal stability of the developed untreated and cross-linked SA-based membranes was investigated by TGA. The resulting thermograms (TG) and their derivatives (DTG) (indicating the dependence of rate of weight change on temperature) for fullerenol, SA-0, SA-5 and cross-linked SA-0^CaCl2^, and SA-5^CaCl2^ membranes are presented in Figure 9.

For all membranes, three distinct weight-loss stages were observed (Figure 9a,b). The first stage of weight loss occurred between 37 and 240 °C, corresponding to the evaporation of the physically absorbed water molecules [45]; the second stage between 240 and 390 °C, corresponded to thermal decomposition of carboxyl and hydroxyl groups; and the third stage over 400 °C was attributed to the degradation of the SA backbone [29]. Compared with the untreated SA-0 and SA-5 membranes, the cross-linking SA-0^CaCl2^ and SA-5^CaCl2^ membranes demonstrated a lower weight loss at the temperature from 240 to 400 °C, which confirmed a deeper cross-linking of polymer chains (more rigid structure). It is also worth noting that the membranes modified by fullerenol (SA-5 and SA-5^CaCl2^) are slightly more thermally stable (less weight loss) when compared to membranes based on pure sodium alginate (SA-0 and SA-0^CaCl2^).

To explain the transport properties of the developed membranes, the swelling degree of the SA-based membranes was studied in water and the separated azeotropic mixture (water/isopropanol (12/88 wt %)) (Table 5).

It was demonstrated that untreated membranes collapsed in water, while the application of the cross-linking method stabilized them in pure water. Swelling degrees in the water (12 wt %)–isopropanol (88 wt %) mixture of untreated SA-0 and modified membranes were close in value due to the small content of water in the studied azeotropic mixture. The cross-linking of membranes led to the more pronounced difference in the swelling degree in the azeotropic mixture for the SA-0^CaCl2^ and SA-5^CaCl2^ membranes due to the more significant differences in the structure. The decrease of swelling degree of the SA-5^CaCl2^ membrane compared to the SA-0^CaCl2^ membrane indicated the cross-linking of SA chains not only by CaCl_2_, but also by fullerenol.

### 3.2. The Development and Investigation of Supported Membranes

An increase in the productivity of the developed dense cross-linked SA-0^CaCl2^ and SA-5^CaCl2^ membranes for the prospective application in industrial dehydration processes is possible by reducing the thickness of the membrane. This problem can be solved by preparing supported membranes, which consist of a thin dense selective layer based on SA and its composite deposited on a porous substrate, to ensure high-performance pervaporation. Porous membranes based on polyacrylonitrile (PAN) were chosen as a substrate, which provided good mechanical strength and did not limit the mass transfer of the components through the membrane.

**Figure 10:**
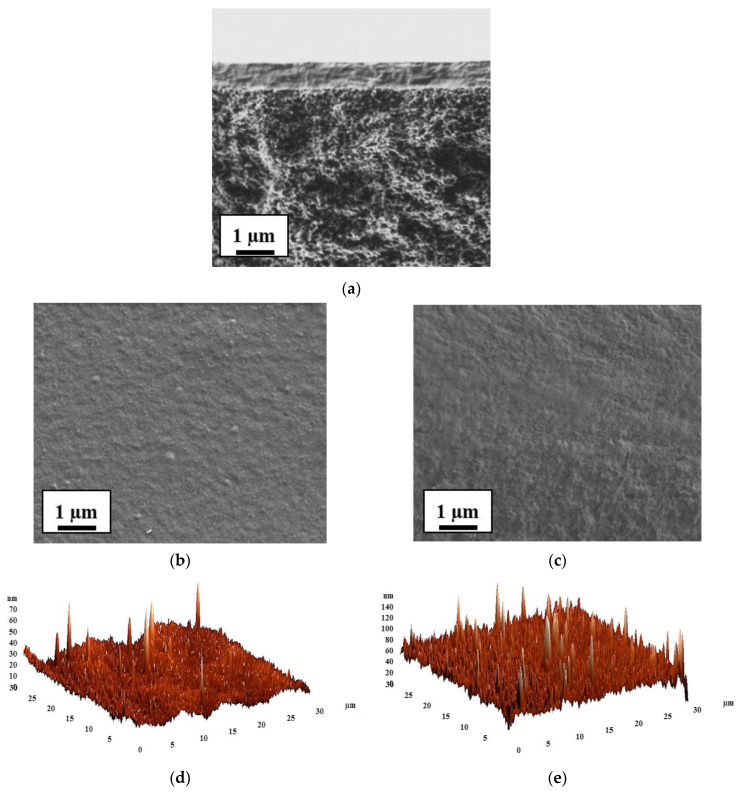
The cross-sectional SEM micrograph (**a**) and SEM micrographs of surface and AFM images of cross-linked supported (**b**,**d**) SA-0/PAN^CaCl2^ and (**c**,**e**) SA-5/PAN^CaCl2^ membranes.

The morphology and the surface topography of the cross-linked SA-0/PAN^CaCl2^ and SA-5/PAN^CaCl2^ membranes were also studied by SEM and AFM. SEM micrographs of the cross-section and surface and AFM images of the SA-0/PAN^CaCl2^ and SA-5/PAN^CaCl2^ membranes are presented in Figure 10. The cross-sectional SEM micrographs of the supported SA-0/PAN^CaCl2^ and SA-5/PAN^CaCl2^ membranes were identical.

The SEM cross-sectional micrograph demonstrates a uniform structure of the top thin dense selective SA–fullerenol (5%) layer and excellent adhesion of it to the porous PAN substrate. Based on the SEM data, the thickness of the selective layer was found to be approximately ~600 nm. This was also demonstrated by the SEM micrographs where the surface of the upper thin dense layer of the supported SA-5/PAN^CaCl2^ membrane was rougher when compared to the SA-0/PAN^CaCl2^ membrane. This was also confirmed by the roughness surface parameters (R_a_ and R_q_), which were calculated for the SA-0/PAN^CaCl2^ and SA-5/PAN^CaCl2^ membranes based on AFM images (Table 6).

It was found that compared to the SA-0/PAN^CaCl2^ membrane, for the SA-5/PAN^CaCl2^ membrane, the root-mean-squared surface roughness (R_q_) and average roughness (R_a_) (Table 6) increased up to 10.8 and 7.6 nm, respectively. This effect was related to the introduction of fullerenol into the SA matrix and could lead to the rise in permeability for the modified membrane. The surface roughness of the cross-linked supported SA-0/PAN^CaCl2^ and SA-5/PAN^CaCl2^ membranes decreased by 2.9 and 6 times when compared to the cross-linked dense SA-0^CaCl2^ and SA-5^CaCl2^ membranes, which was more likely due to the effect of the application of a PAN substrate for a thin dense selective hybrid layer (~600 nm).

**Figure 11:**
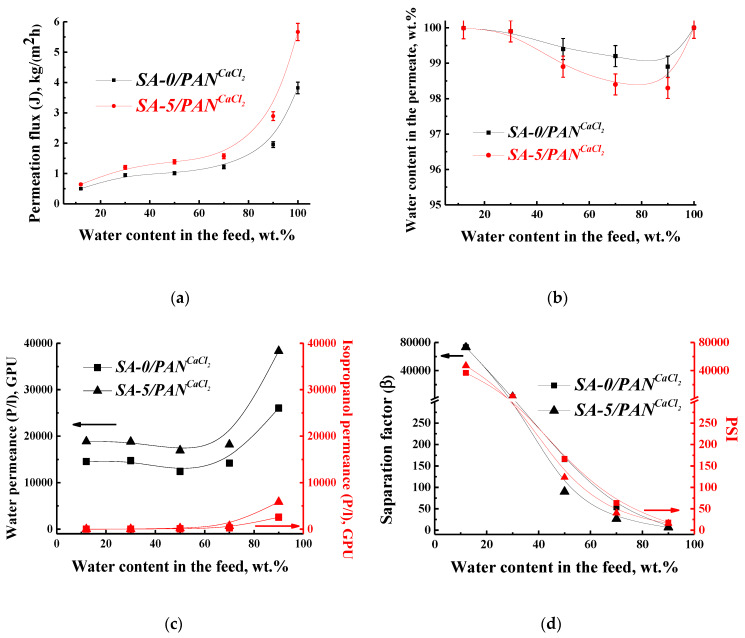
The dependence of (**a**) permeation flux and (**b**) water content in the permeate, (**c**) water and isopropanol permeances, (**d**) separation factor and PSI on water content in the feed during pervaporation of water–isopropanol mixture at 22 °C for cross-linked supported SA-0/PAN^CaCl2^ and SA-5/PAN^CaCl2^ membranes.

To evaluate the transport properties, the supported SA-0/PAN^CaCl2^ and SA-5/PAN^CaCl2^ membranes were tested in the pervaporation separation of water–isopropanol mixture in a wide concentration range (12–100 wt % water). Pervaporation data, in terms of permeation flux, water content in the permeate, component permeances, separation factor, and PSI are presented in Figure 11.

The developed supported cross-linked membranes possessed increased permeation flux (0.5–3.8 kg/(m^2^ h) for SA-0/PAN^CaCl2^ and 0.6–5.7 kg/(m^2^ h) for SA-5/PAN^CaCl2^) compared with dense cross-linked membranes (0.2–0.5 kg/(m^2^ h) for SA-0^CaCl2^ and 0.2–1.2 kg/(m^2^ h) for SA-5^CaCl2^) (Figure 4a). Moreover, the permeation flux of the modified supported SA-5/PAN^CaCl2^ membrane was superior by ~1.5 times to the permeation flux of the SA-0/PAN^CaCl2^ membrane based on the pure polymer (Figure 11a). All developed membranes (dense and supported) had similar values of water content in the permeate. 

Additionally, the supported cross-linked membranes had increased water permeance (14,554–26,034 GPU for SA-0/PAN^CaCl2^ and 18,844–38,338 GPU for SA-5/PAN^CaCl2^) compared with dense cross-linked membranes (4400–5287 GPU for SA-0^CaCl2^and 6884–16,280 for SA-5^CaCl2^). The water permeance for the supported SA-5/PAN^CaCl2^ membrane had the same trend as that of the supported SA-0/PAN^CaCl2^ membrane. These were relatively at the same level of values, and sharply increased at 90 wt % water in the feed, while isopropanol permeance was slightly increased over the entire concentration range (Figure 11c). Additionally, it should be noted that the water permeances for both supported membranes slightly decreased with the rise to 50 wt % water content in the feed, and a decrease in selectivity was observed. This may be due to the plasticizing effect of the membrane in the presence of water, which led to membrane swelling, resulting in non-selective penetration. The separation factor and PSI were larger in value for the SA-5/PAN^CaCl2^ membrane when compared to the SA-0/PAN^CaCl2^ membrane (Figure 11d). This also indicated the effectiveness of the developed modified by fullerenol membrane for the use in pervaporation dehydration. Thus, the developed supported cross-linked SA-5/PAN^CaCl2^ membrane (modified by 5 wt % fullerenol) had the best transport properties (the highest permeation flux and selectivity) for the pervaporation separation of a water–isopropanol mixture in a wide concentration range and is suitable for the promising application in industry.

### 3.3. Comparison of Performance with SA-Based Membranes

Table 7 demonstrates a comparison of the transport properties of SA-5^CaCl2^ and SA-5/PAN^CaCl2^ membranes developed in this work and the SA-based membranes described in the literature for pervaporation dehydration of isopropanol in terms of the permeation flux and the separation factor under conditions close to the present study. Additionally, in the present study, the pervaporation dehydration of isopropanol (12 wt % water) was carried out with the use of commercial membrane PERVAP™ 1201 from Sulzer Chemtech, which was the cross-linked supported membrane used primarily for the dehydration of mixtures up to 80 wt % water [71].

It was demonstrated that the developed dense SA-5^CaCl2^ membrane exhibited the highest separation factor and was slightly inferior in permeation flux when compared to the some modified dense SA-based membranes obtained in previous studies (Table 7). Moreover, the development of the supported SA-5/PAN^CaCl2^ membrane improved both transport parameters, namely, providing the highest separation factor and the permeation flux for the pervaporation separation of an isopropanol–water (88/12 wt %) mixture (Table 7). It was demonstrated that the permeation flux of the developed SA-5/PAN^CaCl2^ membrane was ~23 times higher with the same level of selectivity (separation factor of 73326) compared to the commercial PERVAP™ 1201 membrane in pervaporation for the separation of an azeotropic isopropanol–water (88/12 wt %) mixture at 22 °C (Table 7), which confirmed the promising application of this developed supported SA-5/PAN^CaCl2^ membrane in industry for dehydration processes (e.g., wastewater treatment and purification of chemicals).

## 4. Conclusions

In this study, novel dense and supported mixed matrix membranes based on biopolymer sodium alginate modified by fullerenol were developed. Two types of SA and SA–fullerenol membranes were prepared: untreated (without additional treatment) and cross-linked by CaCl_2_.

For the untreated fullerenol-modified membranes, the formation of the hydrogen bonds between fullerenol and SA was found by FTIR spectroscopy. For the 1.25 wt % CaCl_2_ water solution cross-linked SA and SA–fullerenol (5%) membranes, the organization of polymer chains in the “egg box” structure was demonstrated by NMR and FTIR. Moreover, the introduction of 5 wt % fullerenol in the SA matrix was found to decrease the cross-linking effect of CaCl_2_ and reduce the linked polymer blocks in the SA-5^CaCl2^ membrane.

The modification of the SA matrix by fullerenol greatly enhanced the surface roughness confirmed by SEM and AFM. This effect was more pronounced for the cross-linked SA and SA–fullerenol (5%) membranes. It was also demonstrated that the use of the cross-linking method ensured the stability of the SA and SA–fullerenol (5%) membranes in pure water, which was confirmed by the sorption experiments. The strong cross-linking effect and a more rigid structure of the developed cross-linked membranes were confirmed by TGA data when compared to the untreated membranes.

Transport properties of developed membranes were evaluated in the pervaporation dehydration of isopropanol. For the untreated dense membranes, it was shown that in the pervaporation separation of the azeotropic water (12 wt %)–isopropanol (88 wt %) mixture, the SA membrane modified by 5 wt % fullerenol (SA-5) possessed the highest permeation flux due to the structuring of SA membrane, as confirmed by the NMR data. The cross-linking with CaCl_2_ of SA-0 and SA-5 membranes allows for the application of these membranes in pervaporation for the separation of water–isopropanol mixtures in a wide concentration range (12–90 wt % water) including for pure water penetration. The modified cross-linked dense SA-5^CaCl2^ membrane exhibited the best transport characteristics (higher stability, permeation flux, and selectivity) compared to the untreated SA-5 membrane due to the significant structural changes caused by the introduction of fullerenol into the SA matrix, which acted as the modifier and cross-linker agent with simultaneous application of CaCl_2_. 

To increase the performance of the best modified dense cross-linked SA-5^CaCl2^ membrane for pervaporation dehydration of isopropanol, the novel cross-linked supported membrane consisting of top thin dense selective layer based on SA-fullerenol (5%) composite deposited on a PAN substrate (SA-5/PAN^CaCl2^) was developed. It was shown that the modified supported SA-5/PAN^CaCl2^ membrane was superior in permeation flux ~23 times to the commercial PERVAP™ 1201 membrane (intended for dehydration up to 80 wt.% water) with the same level of selectivity in the pervaporation separation of an azeotropic isopropanol–water (88/12 wt %) mixture. Thus, the SA-5/PAN^CaCl2^ membrane is promising for use in industrial processes of dehydration.

## Figures and Tables

**Table 1:** Developed membranes based on sodium alginate (SA).

Membrane	Type	Thickness, μm	Content of Fullerenol, wt %	Cross-Linking Method
SA-0	dense	25	0	-
SA-3	dense	25	3	-
SA-5	dense	25	5	-
SA-7	dense	25	7	-
SA-10	dense	25	10	-
SA-0^CaCl2^	dense	25	0	1.25 wt % calcium chloride (CaCl_2_)
SA-5^CaCl2^	dense	25	5	1.25 wt % calcium chloride (CaCl_2_)
SA-0/PAN^CaCl2^	supported	0.6	0	1.25 wt % calcium chloride (CaCl_2_)
SA-5/PAN^CaCl2^	supported	0.6	5	1.25 wt % calcium chloride (CaCl_2_)

**Table 2:** Hansen’s Solubility Parameters.

Polymer and Solvents	Hansen Solubility Parameters [MPa^1/2^]				References
	δ_d_	δ_p_	δ_h_	δ_t_	
Sodium alginate	-	-	-	37	[58]
Water	15.5	16	42.3	47.8	[59]
Isopropanol	15.8	6.1	16.4	23.6	

**Table 3:** The crystalline phase content for untreated and cross-linked membranes.

Membranes	Crystalline Phase, %
SA-0	40
SA-5	53
SA-10	41
SA-0^CaCl2^	58
SA-5^CaCl2^	41

**Table 4:** Surface parameters of dense SA and SA–fullerenol membranes.

Membranes	R_a_, nm	R_q_, nm
SA-0	7.2 ± 2	10.2 ± 3
SA-3	25.2 ± 3	33.8 ± 4
SA-5	32.8 ± 3	42.6 ± 4
SA-7	46.9 ± 5	59.7 ± 5
SA-10	67.3 ± 5	84.8 ± 6
SA-0^CaCl2^	11.2 ± 3	15.9 ± 4
SA-5^CaCl2^	45.7 ± 5	59.1 ± 5

**Table 5:** Swelling degree of SA-based membranes in water and the water (12 wt %)–isopropanol (88 wt %) mixture.

Membranes	Swelling Degree in Water (S), %	Swelling Degree in the Azeotropic Mixture (S), %
SA-0	-	15
SA-3	-	13
SA-5	-	12
SA-7	-	14
SA-10	-	19
SA-0^CaCl2^	83	48
SA-5^CaCl2^	77	24

**Table 6:** Surface parameters of the supported SA-0/PAN^CaCl2^ and SA-5/PAN^CaCl2^ membranes.

Membranes	R_a_, nm	R_q_, nm
SA-0/PAN^CaCl2^	3.8 ± 2	5.1 ± 2
SA-5/PAN^CaCl2^	7.6 ± 3	10.8 ± 4

**Table 7:** Comparison of transport properties of SA-based membranes for isopropanol dehydration.

Membranes	Thickness of Selective Layer, µm	Water Content in the Feed, wt %	Temperature, °C	Permeation Flux, kg/(m^2^ h)	Separation Factor (*β*)	Reference
SA-5^CaCl2^	25	12	22	0.240	73,326	This study
SA-5/PAN^CaCl2^	0.6	12	22	0.641	73,326	This study
PERVAP™ 1201	-	12	22	0.028	73,326	This study
Alg-chitosan-wrapped MWCNT (2%)	50	10	30	0.218	6419	[45]
Alg-phosphomolybdic acid (10%)	50	10	30	0.282	9028	[72]
Alg-phosphotungstic acid modified by ammonium carbonate (10%)	50	10	30	0.316	8991	[73]
Alg-3-aminopropyl triethoxysilane (APTEOS)/TEOS (30%)	50	5	30	0.044	17,253	[74]
Alg-gelatin (10%)	45	10	30	0.085	4277	[75]

## Supplementary Materials

The following are available online at https://www.mdpi.com/2073-4360/12/4/864/s1, Figure S1: Schematic representation of a linear chain of alginate with the blocks of munnuronate (M) and guluronate (G), Figure S2: NMR spectra of (**a**) SA-0, (**b**) SA-5, (**c**) SA-10, (**d**) SA-0^CaCl2^, and (**e**) SA-5^CaCl2^ membranes and their decomposition into the corresponding components, Table S1: The chemical shifts of components in the NMR spectra of the developed membranes.
