# Supplementary material for: Novel Mixed Matrix Sodium Alginate–Fullerenol Membranes: Development, Characterization, and Study in Pervaporation Dehydration of Isopropanol
Source: Polymers (Basel). 2020 Apr 9;12(4):864. doi: 10.3390/polym12040864 (PMC7240529; doi:10.3390/polym12040864)
Supplement: Supplementary file 1 [file polymers-12-00864-s001.pdf]

Supplementary material

Nuclear Magnetic Resonance

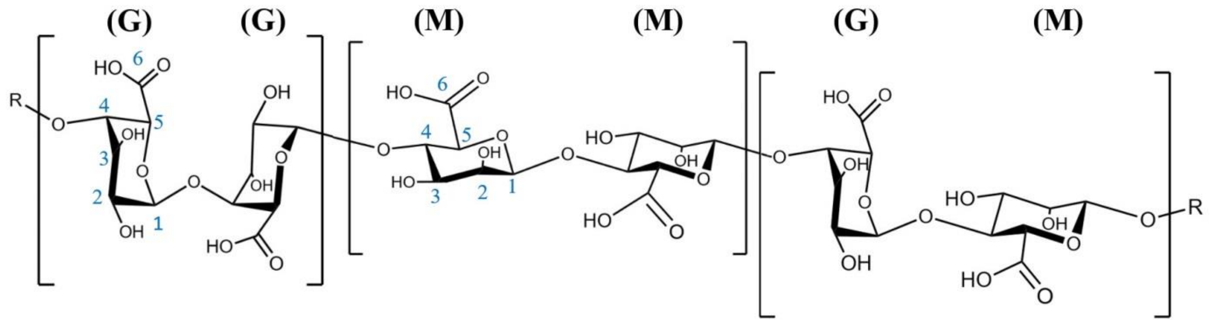

Figure S1. Schematic representation of a linear chain of alginate with the blocks of munnuronate (M) and guluronate (G).

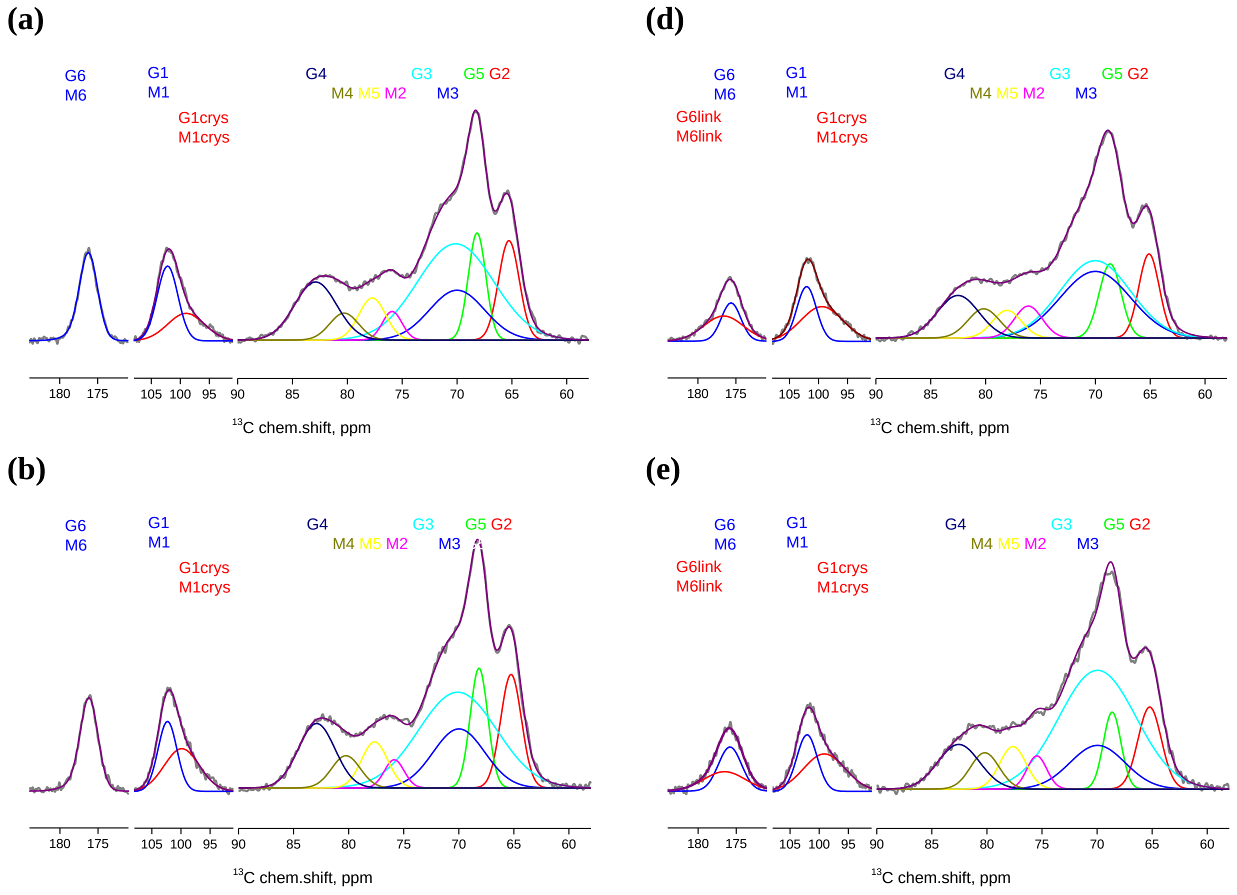

(c)

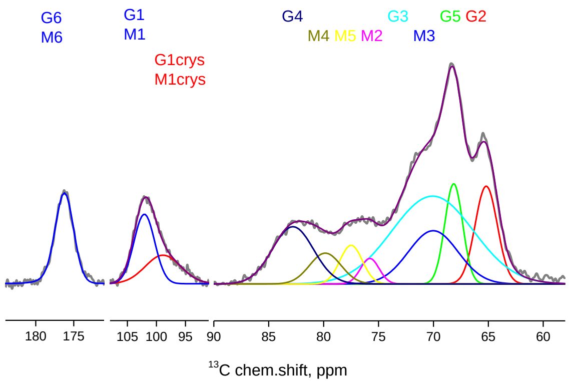

Figure S2. NMR spectra of (a) SA-0, (b) SA-5, (c)SA-10, (d) SA-0^{CaCl2} and (e) SA-5^{CaCl2} membranes and their decomposition into the corresponding components. The spectra demonstrate the peaks of carbon atoms corresponding to Fig. S1 in various positions and in the presence of NMR crystallization and $CaCl_2$ cross-linking.

Table S1. The chemical shifts of components in the NMR spectra of the developed membranes.

Membranes	G2, ppm	G3, ppm	G4, ppm	G5, ppm	M2, ppm	M3, ppm	M4, ppm	M5, ppm
SA-0	65.3	70.1	82.9	68.2	76	70	80.3	77.7
SA-5	65.3	70.1	82.9	68.2	75.9	70	80.2	77.6
SA-10	65.2	70.1	82.8	68.2	75.8	70	79.8	77.5
SA-0^{CaCl2}	65.1	70	82.5	68.7	76.1	70	80.2	78.1
SA-5^{CaCl2}	65.2	70	82.6	68.6	75.5	70	80.2	77.6
